# Supplementary material for: Diffusion-limited association of disordered protein by non-native electrostatic interactions
Source: Nat Commun. 2018 Nov 9;9:4707. doi: 10.1038/s41467-018-06866-y (PMC6226484; doi:10.1038/s41467-018-06866-y)
Supplement: Supplementary file 1 — Supplementary Information [file 41467_2018_6866_MOESM1_ESM.pdf]

Supplementary Information

Diffusion-limited association of disordered protein by non-native electrostatic interactions

Kim *et al*.

This PDF file includes:

Supplementary Figures 1 to 8
Supplementary Tables 1 to 3

a

AviTag — Linker

GHMGMSGLNDIFEAQKIEWHE SSGLVAGGGGSGGGGSGGGGS

UPLSQETFSDLWKLLPENNVLSPLPSQAMDDLMLSPDDIEQWFTEDPGPDC

TAD

b

GSPNRSISPCALQDLLRTLKSPSSPQQQQQVLNILKSNPQLMAAFIKQRTAKYVANQPGMQ

NCBD

c

AviTag — Linker

GSGHMGMSGLNDIFEAQKIEWHE SSGLVAGGGGSGGGGSGGGGS

AQVINTFDGVADYLQTYHKLPDNYITKSEAQALCWVASKGNLADVAPGKSIGGDIFSNR

EGKLPGKSGRTWREADINYTSGFRNSDRILYSSDWLIYKTTDAYQTFTKIR

Barnase

d

GSCGSKKAVINGEQIRSISDLHQTLKKELALPEYYGENLDALWDALTGWVEYPLVLEWRQ

FEQSKQLTENGAESVLQVFREAKAEGADITIILS

Barstar

Supplementary Fig. 1 Protein sequences. **a** Transactivation domain (TAD) of p53 (residue 13 - 61). Biotin was attached to lysine (blue K) in the biotin accepting sequence (AviTag). Glycine-rich spacer (linker) sequence was added between AviTag and TAD to minimize protein-surface interactions. The donor (Alexa 488) and acceptor (Alexa 647) were attached to the cysteine (green C) and unnatural amino acid, 4-acetylphenylalanine (red U) residues at the C- and N-termini of TAD, respectively. **b** NCBD (residue 2059 - 2117). **c** Barnase with AviTag and linker sequences. Glycine was replaced by cysteine (G34C) for labeling the donor (Alexa 488). Histidine was replaced by alanine (H102A) to increase the dissociation rate because binding of wildtype barnase and barstar is effectively irreversible. **d** Barstar. Cysteine was added to the N-terminus of the protein for labeling the acceptor (Alexa 647).

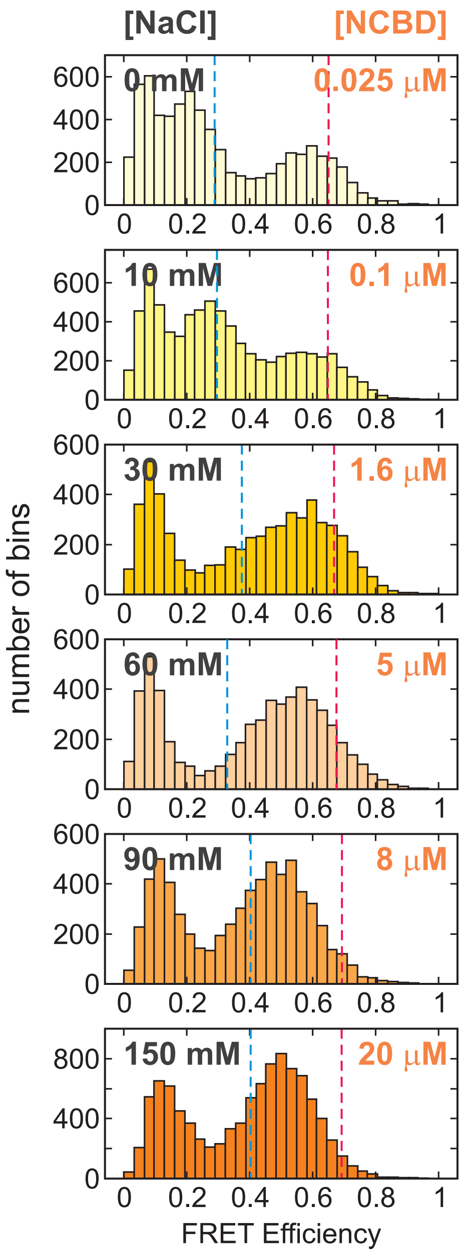

Supplementary Fig. 2 FRET efficiency histograms obtained from freely diffusing TAD and NCBD at various NaCl concentrations. Bin time is 2 ms. The peak near E = 0.1 results from molecules without an active acceptor (donor-only). Blue and red dashed lines indicate the FRET efficiencies of the unbound and bound states, respectively, obtained from the maximum likelihood analysis of the immobilization data in Fig. 2a. Quoted numbers in the FRET efficiency histograms are NaCl (Left) and NCBD (Right) concentrations.

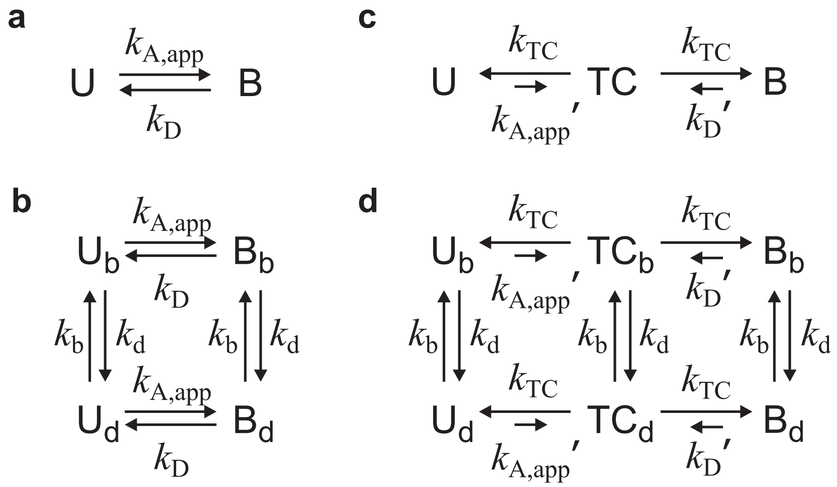

Supplementary Fig. 3 Kinetic models of binding. **a** Two-state model used for the analysis of binding kinetics of barnase and barstar (Fig. 2d and Supplementary Fig. 7 and Supplementary Table 3). **b** Two-state model with acceptor blinking (four-state model) used in the analysis of binding kinetics of TAD and NCBD (Fig. 2c and Supplementary Table 1). **c** Three-state model used for the determination of the lifetime of TC of barnase and barstar binding (Fig. 3d). **d** Three-state model with acceptor blinking (six-state model) used for the determination of the lifetime of TC of TAD and NCBD binding (Fig. 3 and Supplementary Table 2).

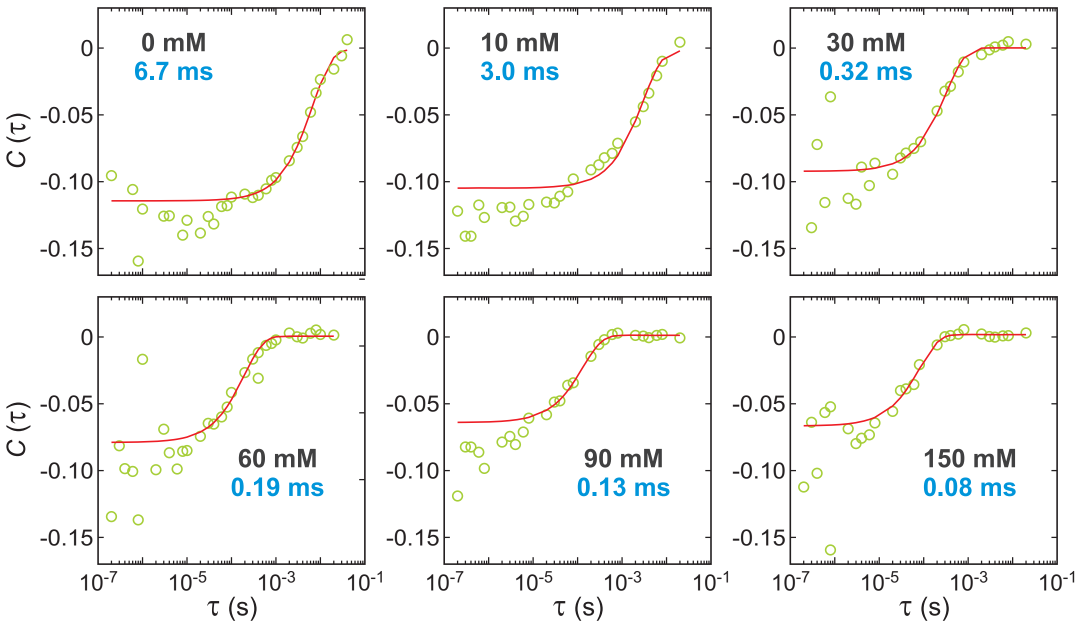

Supplementary Fig. 4 Donor-acceptor cross-correlation analysis. Donor-acceptor cross-correlation functions were calculated for the data of binding of TAD and NCBD in Fig. 2 collected at various NaCl concentrations using equation (7). Red curves are fits to a single exponential function. Fitted parameters can also be found in Supplementary Table 1.

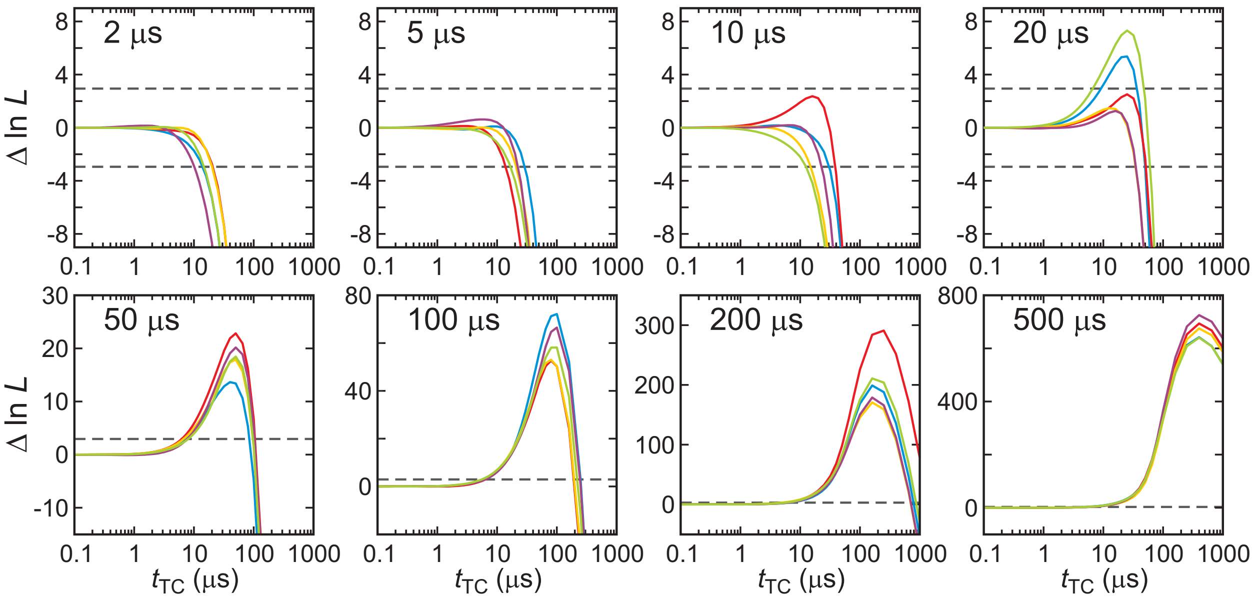

Supplementary Fig. 5 Determination of t_{TC} of simulated photon trajectories. Photon trajectories were simulated with different t_{TC} values. The difference of the log-likelihood function, $\Delta \ln L = \ln L(t_{TC}) - \ln L(0)$ was calculated for five set (curves with different colors) of photon trajectories recolored using the experimental parameters at 0 mM NaCl (Supplementary Table 2).

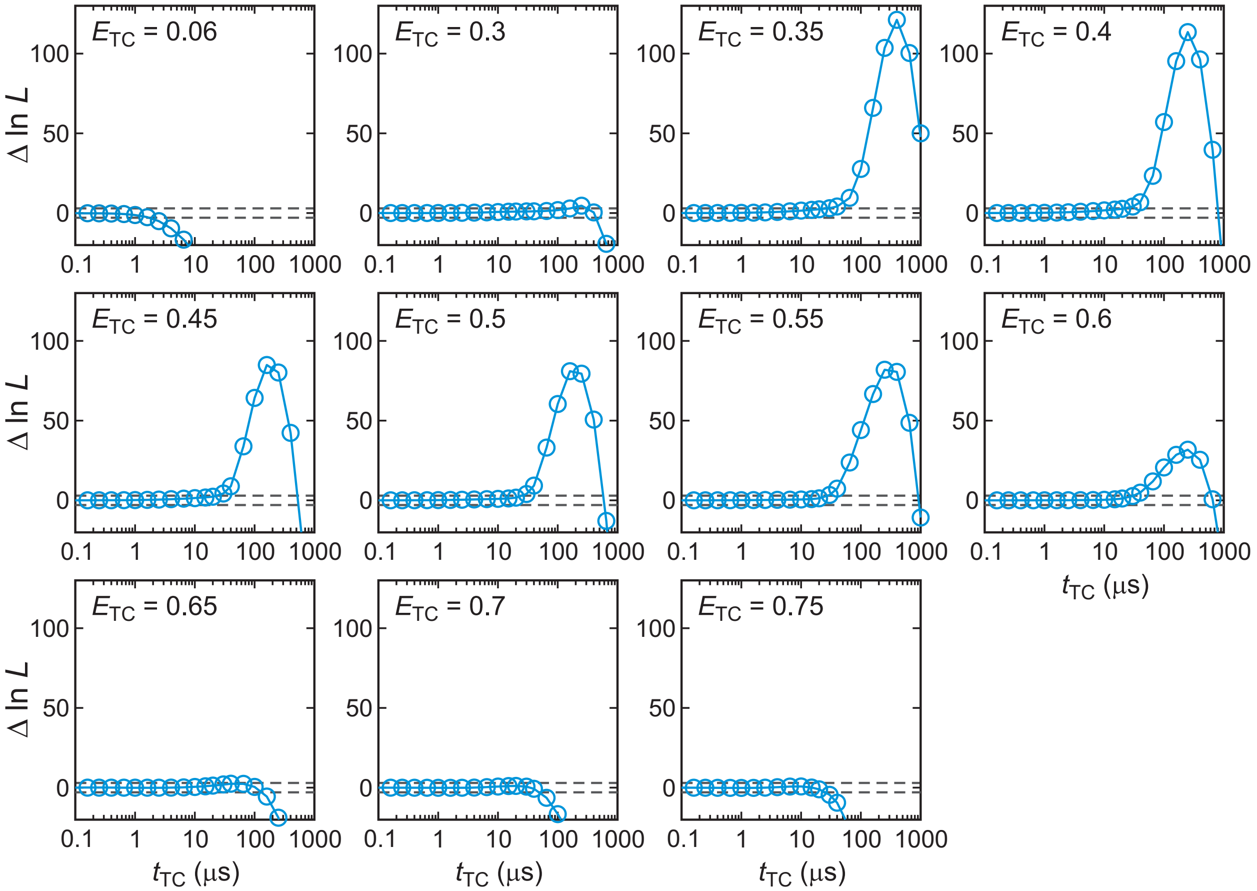

Supplementary Fig. 6 Dependence of t_{TC} on E_{TC} of TAD and NCBD binding. The difference of the log-likelihood function, $\Delta \ln L = \ln L(t_{TC}) - \ln L(0)$ was calculated with different E_{TC} values for the photon trajectories collected at 0 mM NaCl. The maximum of the likelihood is found at E_{TC} ~ 0.35. The accurate value of E_{TC} can be found by maximizing $\ln L(E_{TC}, t_{TC})$ (see Supplementary Table 2).

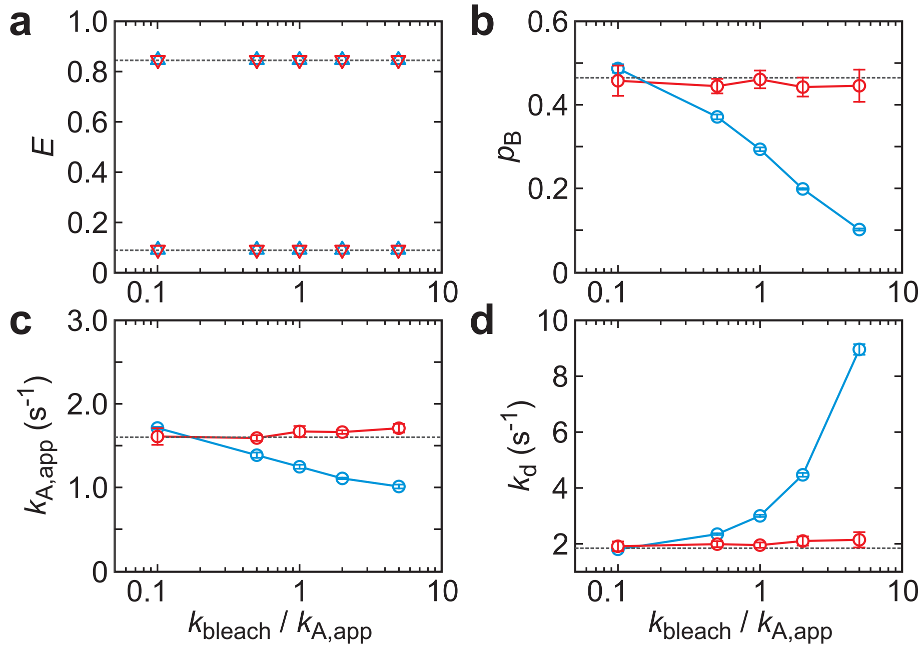

Supplementary Fig. 7 Effect and correction of acceptor bleaching in the binding kinetics of barnase and barstar. Two maximum likelihood analysis results are compared for the recolored photon trajectories of the data measured at 0 mM NaCl. Photon trajectories were recolored with the experimental parameters listed in Supplementary Table 3 (0 mM NaCl) (horizontal dashed lines), and an additional photobleaching rate of the acceptor (k_{bleach}). For each bound state, if a randomly generated bleaching time is shorter than the waiting time, photons after bleaching were recolored with $E = E_d$ (= E_U). The recolored data were analyzed with the two-state model with the bound fraction p_B as a free parameter (blue symbols) or fixed to the value calculated from the fraction of the trajectories beginning with the bound state. Error bars are the standard deviations of the values obtained from the analysis of five recolored data set. **a** FRET efficiencies are very similar in both analyses, indicating that they are hardly affected by acceptor bleaching. On the other hand, when p_B is a free parameter, p_B decreases (**b**), the apparent association rate decreases (**c**), and the dissociation rate increases (**d**) as the photobleaching rate (k_{bleach}) increases. When p_B is fixed, extracted parameters are close to the true values (horizontal dashed lines).

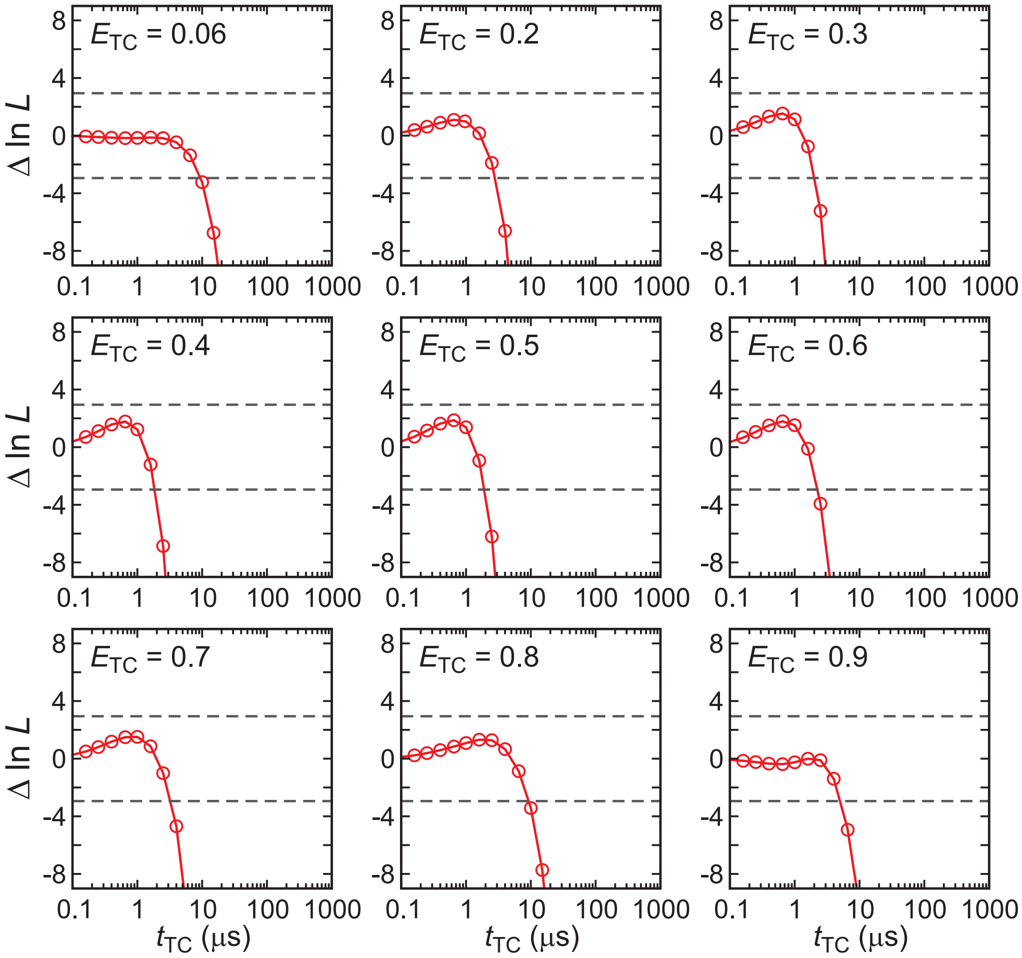

Supplementary Fig. 8 Dependence of t_{TC} on E_{TC} of binding of barnase and barstar. The difference of the log-likelihood function, $\Delta \ln L = \ln L(t_{TC}) - \ln L(0)$ was calculated with different E_{TC} values for the photon trajectories collected at 0 mM NaCl.

Supplementary Table 1 Kinetic parameters of binding of TAD and NCBD obtained from the 4-state (2-state + acceptor blinking) maximum likelihood analysis.

[NaCl] (mM)	0	10	30	60	90	150
[NCBD] (μM)	0.025	0.1	1.6	5	8	20
E_B	0.651 (± 0.001)	0.648 (± 0.001)	0.668 (± 0.002)	0.657 (± 0.002)	0.693 (± 0.002)	0.692 (± 0.003)
E_U	0.289 (± 0.001)	0.296 (± 0.001)	0.375 (± 0.003)	0.347 (± 0.003)	0.403 (± 0.003)	0.403 (± 0.003)
k (ms^{-1})	0.164 (± 0.009)	0.367 (± 0.016)	2.73 (± 0.10)	6.32 (± 0.19)	6.80 (± 0.027)	11.6 (± 0.4)
p_B	0.561 (± 0.018)	0.489 (± 0.014)	0.500 (± 0.007)	0.519 (± 0.006)	0.432 (± 0.007)	0.401 (± 0.008)
k_b (ms^{-1})	12.2 (± 0.6)	14.2 (± 0.8)	11.7 (± 0.7)	8.64 (± 0.49)	9.95 (± 0.53)	8.69 (± 0.52)
$p_b{}^*$	0.912 (± 0.003)	0.912 (± 0.003)	0.932 (± 0.004)	0.957 (± 0.003)	0.949 (± 0.003)	0.964 (± 0.002)
$K_{A,app}$ (ms^{-1})	0.0918	0.180	1.36	3.04	2.94	4.64
k_D (ms^{-1})	0.0719	0.187	1.36	3.28	3.86	6.93
k_A (× 10^9 M^{-1}s^{-1})	3.67	1.80	0.853	0.655	0.367	0.232
K_D (μM)	0.020	0.104	1.60	4.64	10.53	29.9
Donor-Acceptor Cross Correlation, k (ms^{-1})	0.148 (± 0.010)	0.336 (± 0.069)	3.13 (± 0.24)	5.15 (± 0.40)	7.57 (± 0.57)	12.3 (± 1.5)

Errors are standard deviations obtained from the diagonal elements of the covariance matrix calculated at the maximum of the likelihood function.

* At a photon count rate of 100 ms^{-1}.

Supplementary Table 2 Maximum likelihood parameters for the determination of the lifetime of the transient complex (TC).

[NaCl] (mM)	0	10	30	60	90	150
E_B	0.629 (± 0.002)	0.651 (± 0.003)	0.665 (± 0.002)	0.654 (± 0.002)	0.661 (± 0.003)	0.634 (± 0.004)
E_U	0.280 (± 0.001)	0.327 (± 0.003)	0.347 (± 0.002)	0.369 (± 0.002)	0.382 (± 0.002)	0.402 (± 0.004)
k_b (ms^{-1})	176 (± 6)	215 (± 13)	237 (± 12)	378 (± 18)	261 (± 21)	294 (± 31)
p_b*	0.970 (± 0.001)	0.976 (± 0.001)	0.982 (± 0.001)	0.989 (± 0.000)	0.988 (± 0.001)	0.993 (± 0.001)
t_{TC} (μs)	183 (± 16)	175 (± 26)	100 (± 8)	49 (± 6)	46 (± 11)	–
Upper bound t_{TC} (μs)	–	–	–	–	83	83
E_B	0.632 (± 0.002)	0.654 (± 0.003)	0.678 (± 0.003)	0.659 (± 0.002)	0.666 (± 0.003)	–
E_U	0.257 (± 0.001)	0.296 (± 0.003)	0.337 (± 0.002)	0.362 (± 0.002)	0.378 (± 0.003)	–
k_b (ms^{-1})	185 (± 7)	229 (± 13)	241 (± 12)	380 (± 18)	261 (± 21)	–
p_b*	0.970 (± 0.001)	0.977 (± 0.001)	0.982 (± 0.001)	0.989 (± 0.000)	0.988 (± 0.001)	–
t_{TC} (μs)	630 (± 41)	399 (± 45)	143 (± 12)	63 (± 6)	68 (± 15)	–
E_{TC}	0.344 (± 0.003)	0.402 (± 0.006)	0.561 (± 0.009)	0.495 (± 0.015)	0.490 (± 0.020)	–

Errors are standard deviations obtained from the diagonal elements of the covariance matrix calculated at the maximum of the likelihood function. In the upper block, E_B, E_U, k_b, and p_b were obtained by maximizing the likelihood function with the instantaneous transition model (t_{TC} = 0). Then, t_{TC} was determined from the maximum of $\Delta \ln L$ (Fig. 3c). In the lower block, t_{TC} and E_{TC} were determined with other parameters by maximizing the likelihood function.

* At a photon count rate of 100 ms^{-1}.

Supplementary Table 3 Kinetic parameters of binding of barnase and barstar obtained from the two-state maximum likelihood analysis.

[NaCl] (mM)	0	10	30	90
[Barstar] (nM)	20	50	100	200
E_B	0.845 (± 0.000)	0.843 (± 0.001)	0.845 (± 0.000)	0.877 (± 0.000)
E_U	0.089 (± 0.000)	0.094 (± 0.000)	0.110 (± 0.000)	0.147 (± 0.000)
k (s^{-1})	4.74 (± 0.13)	5.70 (± 0.12)	9.78 (± 0.14)	8.84 (± 0.20)
p_B	0.242 (± 0.008)	0.227 (± 0.007)	0.287 (± 0.005)	0.301 (± 0.008)
E_B	0.845 (± 0.000)	0.842 (± 0.001)	0.845 (± 0.000)	0.876 (± 0.000)
E_U	0.089 (± 0.000)	0.094 (± 0.000)	0.110 (± 0.000)	0.147 (± 0.000)
k (s^{-1})	3.44 (± 0.11)	3.36 (± 0.13)	6.30 (± 0.09)	5.91 (± 0.14)
p_B	0.465 (± 0.040)	0.581 (± 0.043)	0.689 (± 0.034)	0.694 (± 0.038)
$k_{A,app}$ (s^{-1})	1.60	1.95	4.34	4.10
k_D (s^{-1})	1.84	1.41	1.96	1.81
k_A ($\times 10^8$ $M^{-1}s^{-1}$)	0.800	0.390	0.434	0.205
K_D (nM)	23	36	45	88

Errors are standard deviations obtained from the diagonal elements of the covariance matrix calculated at the maximum of the likelihood function. In the upper block, parameters were determined with p_B as a free parameter. In the lower block, parameters were determined by fixing p_B to the bound fraction obtained from the fraction of the trajectories beginning with the bound state.
